# Evaluation of a potent LpxC inhibitor for post-exposure prophylaxis treatment of antibiotic-resistant *Burkholderia pseudomallei* in a murine infection model

**DOI:** 10.1128/aac.01295-24

**Published:** 2024-12-13

**Authors:** Henry S. Heine, Bret K. Purcell, Clayton Duncan, Lynda Miller, John E. Craig, Amanda Chase, Lynne Honour, Michael Vicchiarelli, George L. Drusano, Pei Zhou

**Affiliations:** 1 Institute for Therapeutic Innovation, University of Florida 3463 , Orlando, Florida, USA; 2 Valanbio Therapeutics Inc., Raleigh, North Carolina, USA; 3 Department of Biochemistry, Duke University School of Medicine 12277 , Durham, North Carolina, USA; Bill & Melinda Gates Medical Research Institute, Cambridge, Massachusetts, USA

**Keywords:** melioidosis, *B. pseudomallei*, pneumonia, LpxC, LPC-233

## Abstract

LPC-233 (a.k.a. VB-233) is a potent antibiotic targeting the essential enzyme LpxC in Gram-negative bacteria. We present herein the pharmacokinetics and pharmacodynamics data of LPC-233 for treating murine infections caused by *Burkholderia pseudomallei*, a potential biodefense pathogen. A range of doses was evaluated in a post-aerosol exposure model of *B. pseudomallei*-infected mice. After the aerosol challenge with the *B. pseudomallei* strain K96243, treatment was initiated with 10, 30, or 90 mg/kg of LPC-233 orally every 12 h (q12h) or 90 mg/kg intraperitoneally q12h for 14 days. A vehicle-control arm and a positive-control arm consisting of one of the recommended standards of care, ceftazidime (150 mg/kg, q6h) injected subcutaneously, were included. LPC-233 significantly and dose-dependently rescued mice from *B. pseudomallei* infection in comparison with the vehicle (*P* < 0.0001). At dose levels of 30 mg/kg or higher, the survival rate with LPC-233 was significantly higher than that from the ceftazidime arm (*P* range: 0.001–0.05). LPC-233 reversed the murine body weight loss caused by the *B. pseudomallei* infection more rapidly than ceftazidime did, suggesting that it is a faster-acting antibiotic in this dosing regimen. Despite the outstanding survival advantage of LPC-233 over ceftazidime, no significant differences in tissue burdens (liver, lung, spleen, and blood) were observed among any of the treatment groups surviving to the termination of the experiment, suggesting that similar to commercial antibiotics, LPC-233 treatment for lethal *B. pseudomallei* infection may likely require both an acute phase of intensive treatment and an eradication phase of prolonged treatment.

## INTRODUCTION

*Burkholderia pseudomallei*, the etiological Gram-negative bacterial agent of life-threatening melioidosis, is estimated to cause ~89,000 deaths worldwide each year (1). Although *B. pseudomallei* is naturally found in contaminated soil and water in Southeast Asia, Australia, Latin America, and parts of Africa, it is also considered a potential bioterrorism threat, as an intentional aerosol release of *B. pseudomallei* is sufficient to cause melioidosis. The untreated mortality from acute melioidosis can approach 100%. Even with early and aggressive treatment, this disease can also manifest as a slow, debilitating chronic infection. There are no Food and Drug Administration-approved antibiotic indications for *B. pseudomallei* infections. The current practice for treating melioidosis involves an acute phase of intravenous antimicrobial therapy (typically ceftazidime administered every 6–8 h or meropenem administered every 8 h) for 2–8 weeks, followed by an eradication phase of oral antimicrobial therapy (trimethoprim-sulfamethoxazole taken every 12 h or amoxicillin/clavulanic acid taken every 8 h) for 3–6 months. Unfortunately, many clinical strains have demonstrated resistance to these off-label antibiotics (2–4).

Similar to that in other Gram-negative bacteria, the outer leaflet of the outer membrane of *B. pseudomallei* is enriched with lipopolysaccharide (LPS), which is anchored into the bacterial membrane through a penta-acylated disaccharide moiety (5) known as lipid A. Constitutive lipid A biosynthesis is required for bacterial viability and fitness in the human host (6–9); hence, the essential enzymes involved in lipid A biosynthesis, such as the UDP-3-*O*-(*R*-3-hydroxymyristoyl)-*N*-acetyl-glucosamine deacetylase (LpxC), have become attractive targets to create novel classes of small molecule inhibitors as antibacterial agents specific to Gram-negative bacterial pathogens, including *B. pseudomallei*.

Extensive research (8–12) over the last two decades shows that potent LpxC inhibitors display an outstanding bactericidal effect. Virtually, all Gram-negative bacteria are sensitive to LpxC inhibition *in vivo*, and LpxC inhibitors are not inactivated by common resistance mechanisms, such as extended-spectrum beta-lactamases (ESBL) or carbapenemases. Recently, Duke University and Valanbio Therapeutics, Inc. reported a lead LpxC inhibitor, called LPC-233 (a.k.a. VB-233), which is potently and broadly bactericidal against >280 clinical Gram-negative bacterial strains *in vitro*, has high oral bioavailability and broad tissue distribution, and is highly efficacious in multiple murine bacterial infection models (13). Most importantly, although dose-limiting cardiovascular adverse effects have halted the development of two advanced LpxC inhibitors, ACHN-975 (Achaogen) and RC-01 (Recida Therapeutics), LPC-233 did not show the same liabilities in preclinical animal studies, supporting its further development toward clinical studies.

In this research, we report the outstanding *in vitro* activity of LPC-233 against a collection of 30 clinical strains of *B. pseudomallei* with MICs ranging from ≤0.008 to 0.12 µg/mL. Furthermore, oral administration of LPC-233 demonstrates superior efficacy and faster recovery from infection-induced body weight loss over one of the recommended standards of care antibiotic, ceftazidime, in a murine post-exposure prophylaxis treatment model of *B. pseudomallei* infection. The results highlight LPC-233 as a promising oral treatment to replace the intravenous intensive phase antimicrobial therapy.

## RESULTS

### Antibiotic activity of LPC-233 against clinical strains of *B. pseudomallei*

LPC-233 demonstrated broad-spectrum antibiotic activity against 285 strains of clinical Gram-negative pathogens (13). However, its effectiveness against *B. pseudomallei* remained unknown. Therefore, we evaluated the activity of LPC-233 against a collection of 30 strains of *B. pseudomallei* using the standard CLSI broth microdilution assay (14). This collection of strains is representative of the geographic distribution across Southeast Asia and Australia. The majority of these strains are clinical isolates that reflect many of the resistance profiles encountered in the melioidosis “frontline” antibiotic therapies. Ceftazidime was used as the reference compound. We found that LPC-233 exhibited exceptional antibiotic activity against all 30 strains of *B. pseudomallei in vitro*, with minimal inhibitory concentrations (MICs) ranging from ≤0.008–0.12 µg/mL (Table S1). The MIC_50_ and MIC_90_ values were ≤0.008 and 0.03 µg/mL, respectively. In comparison, the frontline therapeutic ceftazidime had MIC values ranging from 0.5 to >16 µg/mL against these strains, with the MIC_50_ and MIC_90_ values of 2 and 8 µg/mL, respectively (Table S1). It is interesting to note that the *B. pseudomallei* strain H4609e has a much higher MIC value of 0.12 µg/mL in comparison with other *B. pseudomallei* strains, such as K96243, 406e, and H942dii, all having MIC values of less than 0.008 µg/mL. Resistance to LpxC inhibitors occurs most frequently in FabZ involved in fatty acid biosynthesis and occasionally in LpxC (15–17). The FabZ enzyme in H4609e shows no sequence variation from those in the highly susceptible strains of K96243, 406e, and H942dii (Fig. S1). The LpxC enzymes in *B. pseudomallei* strains are polymorphic at positions 25 (either E or D) and 153 (either R or K), both of which are located on the protein surface and away from the active site, and are not engaged with substrate or inhibitor interactions (Fig. S2). Furthermore, the LpxC enzyme of strain H4609e has an identical sequence to that of the highly susceptible strain H942dii, indicating that the LpxC enzyme of strain H4609e is not the source of its higher MIC value (Fig. S2). Since strain H4609e also shows a very high MIC value for ceftazidime (MIC > 16 µg/mL), which is an antibiotic of an entirely different class from LPC-233, it is likely that the elevated MIC values of H4609e for both LPC-233 and ceftazidime are due to mutation or overexpression of efflux pumps. Further investigation is needed to elucidate the resistance mechanism in H4609e.

Overall, the outstanding antibiotic activity of LPC-233 against *B. pseudomallei* coupled with its previously reported safety profile indicated that it could potentially be an efficacious therapeutic for treating melioidosis.

### LPC-233 shows high penetration into the lung tissue

Our previous studies demonstrated a broad tissue distribution of LPC-233 in mice, including kidney, liver, and muscle tissues (13), but the bioavailability of LPC-233 in the lung was not evaluated. As the lung is the site of infection through aerosol exposure to *B. pseudomallei*, it is important to determine whether the source-site infection can be adequately controlled. A previous evaluation of other antibiotics demonstrated that lung infection may alter the lung epithelial lining fluid (ELF) penetration (18), suggesting that the penetration of LPC-233 during infection needed to be examined. To address this knowledge gap, we measured the levels of LPC-233 in plasma and ELF from samples of BALB/c mice (female, 6–8 weeks old, with an average weight of 19.8 g) infected through aerosol exposure of *B. pseudomallei* (strain K96243). At 48 h post-bacterial challenge, a single dose of LPC-233 formulated in 20% (w/v) Captisol was administered to infected mice at dosing levels of 10, 30, and 100 mg/kg for oral (PO) delivery or at 100 mg/kg for intraperitoneal (IP) delivery. An untreated arm was also included as the bacterial infection control. Blood and ELF samples were collected at 0.25, 0.5, 1, 2, 4, and 6 h post-dosing. All samples were processed and quantitated by liquid chromatography–tandem mass spectrometry (LC–MS/MS) and used for pharmacokinetics (PK) analysis and modeling.

For this infected PK study, the aerosol exposure target was 25 × LD_50_ (1,900 CFU/mouse; inhaled LD_50_ is 76 CFU/mouse), and the actual exposures were 6.3 × and 5.9 × LD_50_s for the two runs (average 4.7 × 10^2^ CFUs/mouse). Enumeration data from the blood, lung, liver, and spleen of the infection control animals verified that *B. pseudomallei* established infection 48 h after the aerosol challenge. The level of infection was consistent with previous *B. pseudomallei* infection studies in the murine model (19).

**Fig 1:**
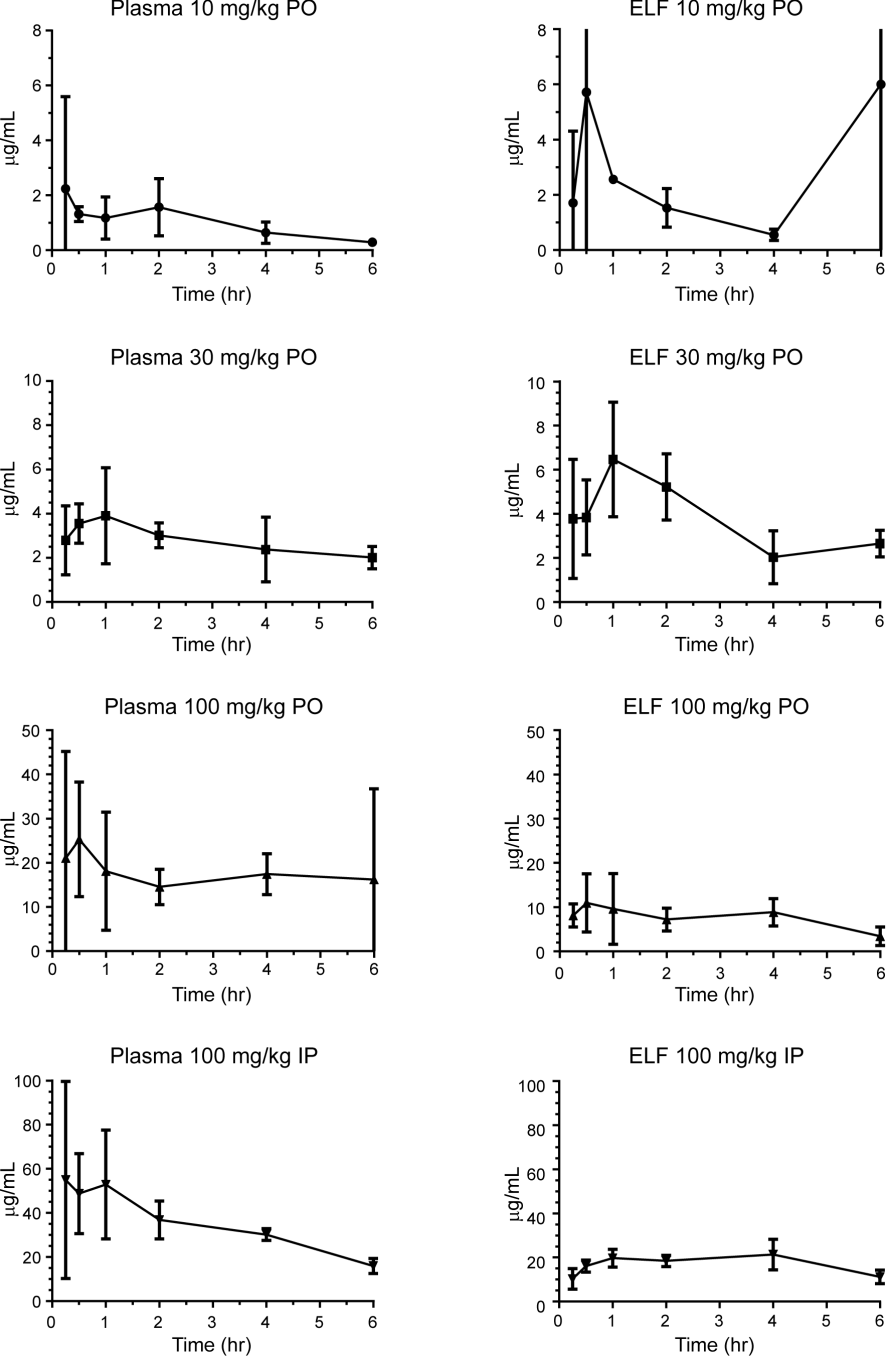
LPC-233 plasma and epithelial lining fluid (ELF) pharmacokinetics. Single doses of LPC-233 were administered 48 h after *B. pseudomallei* aerosol challenge. Blood and ELF were collected from four treated animals after CO_2_ asphyxiation at 0.25, 0.5, 1, 2, 4, and 6 h post-dosing. Blood samples were collected in K_2_-EDTA tubes. Plasma was collected by immediate centrifugation (3000×*g* for 5 min). Lung ELF was obtained by broncho-alveolar lavage with physiological saline. Samples were processed for LC–MS/MS. Data are presented as the mean ± standard deviation.

During the collection of blood samples for plasma, no hemolysis was noted. The collected ELF volumes were within the expected range. The analysis of the PK results (Fig. 1; Table 1) revealed that the LPC-233 concentrations in the infected animals showed a dose response in both AUC (area under the curve) and C_max_ for plasma and ELF with the oral doses. The 100 mg/kg IP dose resulted in a 2-fold increase in AUC and C_max_ compared to the oral 100 mg/kg dose group (Table 1). Interestingly, LPC-233 had greater ELF penetration relative to plasma levels. In fact, the drug levels in the ELF samples exceeded the corresponding plasma levels at the 10 and 30 mg/kg PO doses (Fig. 1). Importantly, at dosing levels of 30 mg/kg or above, a significant concentration of the drug (0.1–0.4 μg/mL) could be detected in the ELF samples even at 6 h post-administration.

**TABLE 1:** Calculated LPC-233 plasma and epithelial lining fluid (ELF) pharmacokinetic values: 0–6 h^*b*^

Group^*a*^	Plasma 10 mg/kg PO	Plasma 30 mg/kg PO	Plasma 100 mg/kg PO	Plasma 100 mg/kg IP	ELF 10 mg/kg PO	ELF 30 mg/kg PO	ELF 100 mg/kg PO	ELF 100 mg/kg IP
Inf AUC	5.6	16	99	197	14	21	44	104
Inf C_max_	2.2	3.9	25	55	6	6.5	11	21
Inf fT > MIC	≥6	≥6	≥6	≥6	≥6	≥6	≥6	≥6
% Penetration ELF					250%	131%	44.4%	52.8%

^*a*^ AUC = area under the curve from 0 h to 6 h (mg*h/L); C_max_ = maximum concentration of drug (µg/mL); fT > MIC = free time above MIC (h); Inf = infected.

^*b*^ The LPC-233 MIC_90_ for *B. pseudomallei* is 0.03 µg/mL. AUC in mg•h/L, C_max_ in mg/L, and fT > MIC in h.

As our infected PK study was undertaken to guide the design of therapeutic regimens in the murine challenge model, we were pleased to find that the drug concentrations remained above the MIC in both plasma and ELF at all doses (Table 1). These data also indicated that both plasma and ELF clearance were reduced relative to other antibiotics in mice, particularly beta-lactams (20), indicating that LPC-233 might be a more effective therapeutic solution than these antibiotics. Modeling of the data collectively for the 100 mg/kg dose from all of the PK data yielded AUC_plasma_ (0–48 h) of 398.6 mg*h/L and AUC_ELF_ (0–48 h) of 334.6 mg*h/L, resulting in an estimate of 83.9% ELF penetration. Clearance was calculated to be 0.88 L/h/kg. Considering the MIC_90_ value (0.03 µg/mL) of LPC-233 against *B. pseudomallei* (Table S1), at the 100 mg/kg dose, the plasma concentration of LPC-233 remained above MIC_90_ over a 24 h period. Our previous studies have shown that LPC-233 is highly plasma bound (with ~96.4% bound to the mouse plasma protein) (13). However, the presence of 50% mouse plasma only modestly shifted the MIC values of LPC-233 against *Escherichia coli*, *Klebsiella pneumoniae*, and *Pseudomonas aeruginosa* by 4–9-fold, far less than the predicted ~30-fold shift based on the analysis of the plasma protein unbound fraction (13). Using a conservative estimation of a 10-fold shift of the MIC_90_ value (10 × 0.03 µg/mL) against *B. pseudomallei*, LPC-233 maintained sufficiently high plasma and ELF concentrations at 4–6 h post 30 mg/kg PO delivery (2.0–2.4 μg/mL for plasma and 2.0–2.7 μg/mL for ELF), 6 h post 100 mg/kg PO delivery (16 µg/mL for plasma and 3.5 µg/mL for ELF), and 6 h post 100 mg/kg IP delivery (16 µg/mL for plasma and 11 µg/mL for ELF), indicating that the 30–100 mg/kg doses would be sufficient to maintain therapeutic antibiotic levels in both plasma and ELF for an extended period.

### Efficacy of LPC-233 in the acute *B. pseudomallei* lung infection model in mice

We next evaluated the effectiveness of LPC-233 in rescuing mice from lethal lung infection due to aerosol exposure of *B. pseudomallei*. The *B. pseudomallei* strain K96243 was used for this study. The entire efficacy study was completed in two separate runs, with the actual challenge doses of approximately 87 × LD_50s_ (6,620 CFU/mouse) for aerosol run #1 and 76 × LD_50_s (5,790 CFU/mouse) for aerosol run #2. The study contained six groups, including a negative control group with no treatment, a positive control group with subcutaneous administration (SC) of ceftazidime at 150 mg/kg q6h (600 mg/kg/day), and four LPC-233 treatment groups consisting of IP administration of LPC-233 at 90 mg/kg q12h (180 mg/kg/day) and oral administration of LPC-233 at 90 mg/kg q12h (180 mg/kg/day), 30 mg/kg q12h (60 mg/kg/day), and 10 mg/kg q12h (20 mg/kg/day). Each of these groups included 10 mice, with five mice from each of the two aerosol runs. Treatment started 24 h post-aerosol exposure of *B. pseudomallei* and lasted 14 days, mimicking the intensive phase of the clinical treatment. The mice were observed for an additional 29 days to evaluate post-treatment survival and clearance of bacterial infection.

**Fig 2:**
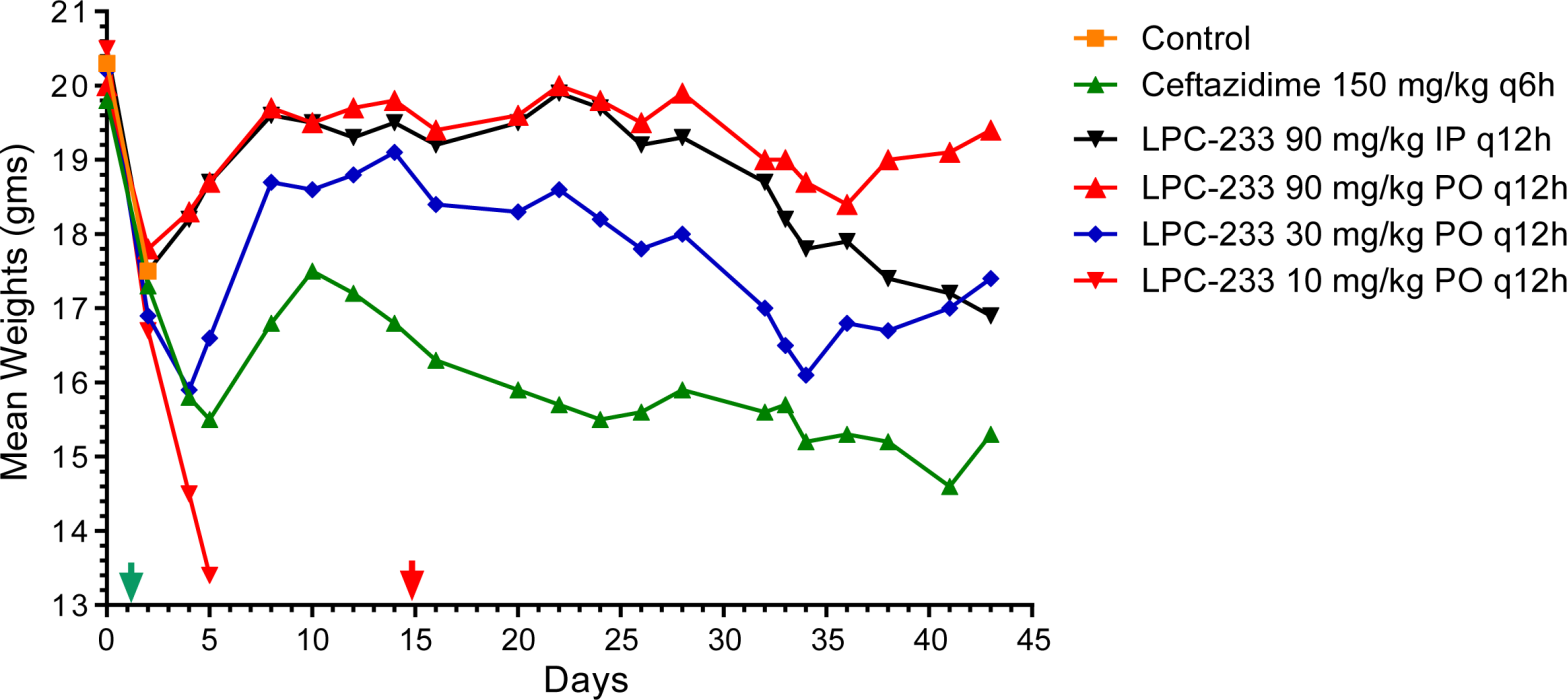
Group mean body weights of *B. pseudomallei*-infected mice in different treatment groups. Mice were weighed by cage group immediately prior to aerosol challenge, and then every 2 days throughout the study. Each point represents the mean of surviving animals in each treatment group. The green and red arrows indicate the start and end of dosing, respectively.

Infection of *B. pseudomallei* caused rapid body weight loss. The mean body weights of all groups declined during the first 48 h after the challenge (Fig. 2). The average body weights in the vehicle control and LPC-233 10 mg/kg groups did not recover prior to all animals succumbing to disease. The average weights of the animals in the ceftazidime 150 mg/kg q6h positive control group began to recover after Day 5, reflecting the therapeutic effect of ceftazidime. This trend also occurred in LPC-233 groups at dosing levels of 30 mg/kg q12h or higher. The mean body weights began to recover sooner than the ceftazidime group, with the turning points of the 30 mg/kg q12h (oral) group occurring at Day 4 and those of the two 90 mg/kg q12h (oral and IP) groups happening at Day 2. These observations suggest that the effect of LPC-233 was exposure dependent, and LPC-233 likely acted faster and more efficiently than ceftazidime.

It is worth noting that even with the initial recovery, the average body weight of the ceftazidime group started to decline again at Day 11 during treatment, indicating that ceftazidime at 150 mg/kg q6h was insufficient to control the *B. pseudomallei* infection during this period. In comparison, the average body weight in the LPC-233 group at 30 mg/kg q12h only declined again after treatment stopped at Day 15, whereas the average body weight in the 90 mg/kg IP group did not decline until 10 days into the post-treatment period (Day 25), and the 90 mg/kg PO group maintained a steady average weight to the end of the study. These results indicate that LPC-233 at doses of 30 mg/kg q12h or higher more effectively suppressed *B. pseudomallei* infection than ceftazidime both during the treatment and observation phases.

**Fig 3:**
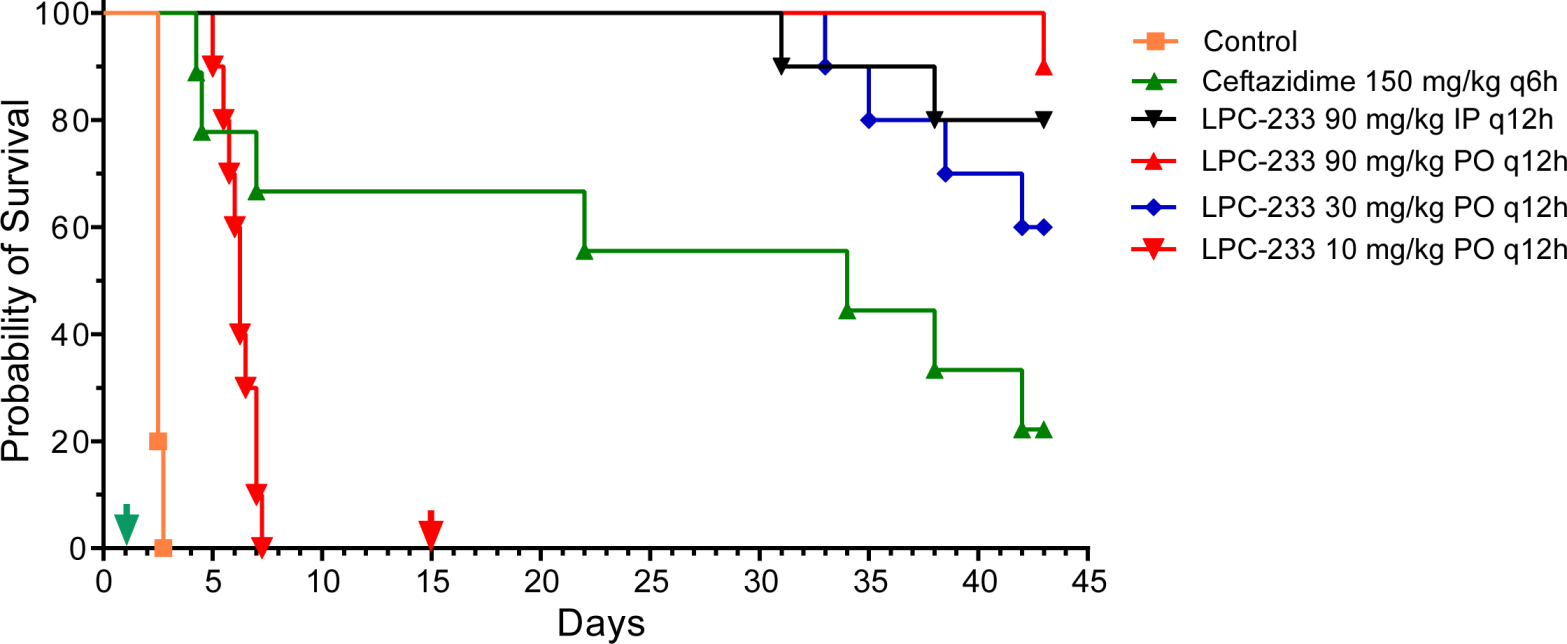
Efficacy of LPC-233 in rescuing mice from lethal *B. pseudomallei* infection. The cohort size for statistical evaluation was 10 mice. Mortality was assessed and recorded every 6 h during the antibiotic administration phase of the study (14 days starting from 24 h post-challenge) and at least twice daily thereafter. The experiment was terminated at Day 43. The green and red arrows indicate start and end of dosing, respectively.

This conclusion is further supported by mouse survival curves (Fig. 3 and statistics in Table 2). Times to death in all treatment groups were significantly different from that of the vehicle control group (*P* < 0.0001). All mice in the vehicle group (negative control) succumbed to death within 3 days post-aerosol exposure of *B. pseudomallei*, whereas SC treatment with ceftazidime at 150 mg/kg q6h achieved 70% survival during the treatment phase and 20% survival at the end of the 29-day observation phase. Oral dosing of LPC-233 at 10 mg/kg q12h did not support survival, but the median time to death was extended compared to the control group (6.3 vs. 2.5 days). The LPC-233 treatment groups at dosing levels of 30 mg/kg q12h (oral) and 90 mg/kg q12h (oral and IP) maintained 100% survival during the treatment phase and achieved the final survival rates at the end of the observation period of 60% for 30 mg/kg q12h oral treatment, 80% for 90 mg/kg q12h IP treatment, and 90% for 90 mg/kg q12h oral treatment. All of the LPC-233 treatment groups at 30 mg/kg q12h or higher demonstrated times to death significantly greater than that of the ceftazidime group (*P* range 0.001–0.05). However, there was no statistical significance in the survival advantage for the two 90 mg/kg groups over the 30 mg/kg group or between the IP and PO routes of the two 90 mg/kg groups (Table 2).

**TABLE 2:** Survival log-rank (Mantel–Cox) test

Survival	Ceftazidime	VB233 90 mg/kg IP	VB233 90 mg/kg PO	VB233 30 mg/kg PO	VB233 10 mg/kg PO
20% Captisol	<0.0001^*a*^	<0.0001^*a*^	<0.0001^*a*^	<0.0001^*a*^	<0.0001^*a*^
Ceftazidime		0.009^*a*^	0.001^*a*^	0.05^*a*^	0.008^*a*^
VB233 90 mg/kg IP			0.5	0.4	<0.0001^*a*^
VB233 90 mg/kg PO				0.1	<0.0001^*a*^
VB233 30 mg/kg PO					<0.0001^*a*^

^*a*^ Significant.

**Fig 4:**
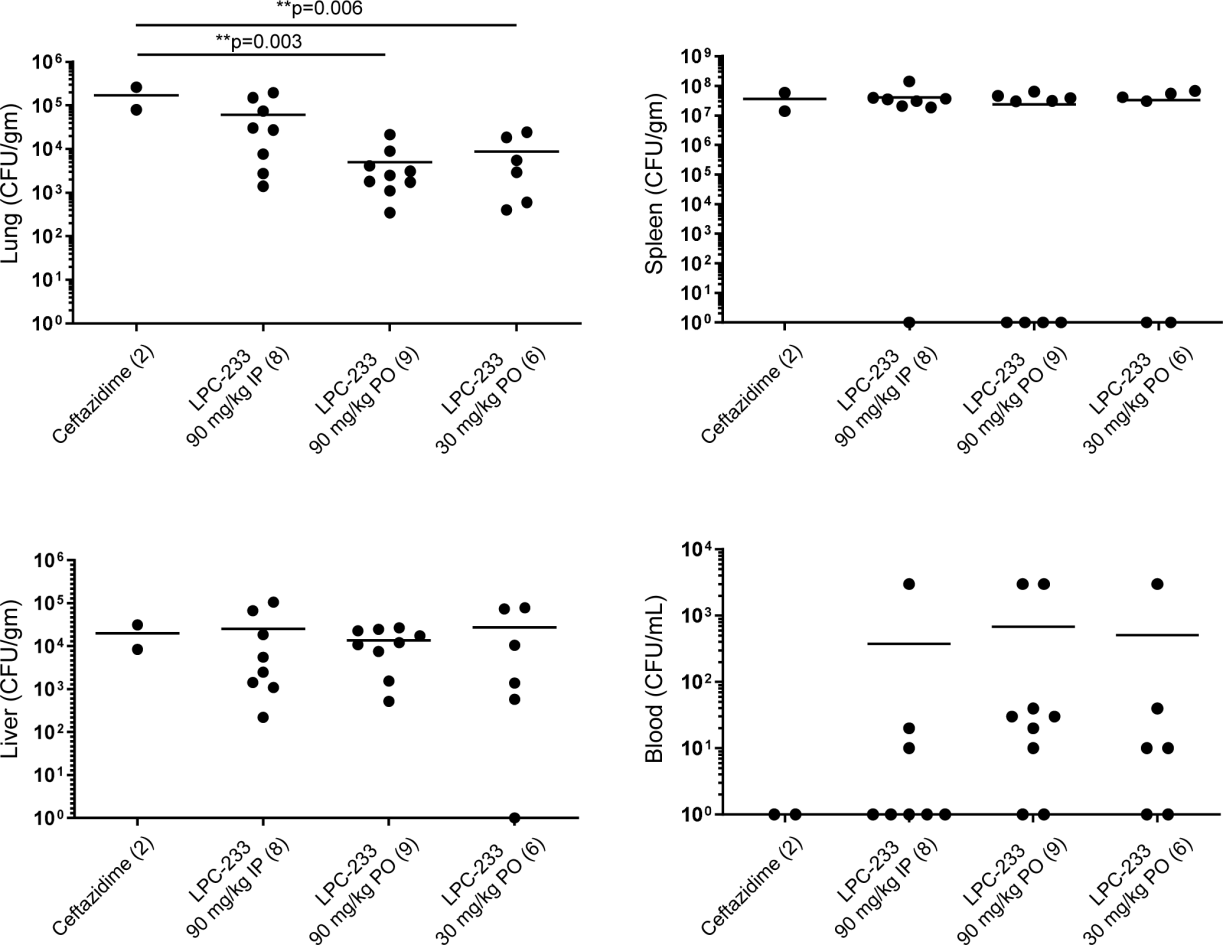
*B. pseudomallei* tissue burdens of mice surviving to study termination. Each point represents the bacterial load in the blood and tissues recovered from animals surviving to study termination. The line represents the mean for each tissue/treatment. Statistical analysis: Bonferroni multiple comparison test with statistically significant comparisons (*P* < 0.05) labeled. **P < 0.01.

Since both body weight loss and increased death rates of mice during the observation period indicated incomplete bacterial elimination, we analyzed the bacterial burdens at the termination of the experiment (Day 43) (Fig. 4). The mean bacterial loads for the livers and spleens were not significantly different among the treatment groups. Treatment with LPC-233 at 90 mg/kg PO or 30 mg/kg PO led to a reduction of CFUs in the lungs with significantly different bacterial burdens compared to the ceftazidime-treated animals. Although a significant difference from the LPC-233 treatment group at 90 mg/kg IP was not observed, oral treatments trended lower. Blood burdens were the most variable, with no significant differences among the treatment groups (*P* = 1).

Taken together, our results indicate that oral or IP deliveries of LPC-233 at dosing levels of 30 mg/kg q12h (60 mg/kg/day) or higher are more effective than ceftazidime at 150 mg/kg q6h (600 mg/kg/day) (SC) in rescuing mice from the lethal *B. pseudomallei* infection in the acute phase of intensive treatment. Although none of the LPC-233 groups completely eliminated *B. pseudomallei* in the mice, further extension of the treatment period or optimization of the dosing regimen may achieve a complete clearance of *B. pseudomallei* infection.

## DISCUSSION

Ceftazidime is one of the frontline antibiotics used in the intensive phase of treatment for *B. pseudomallei* infection and serves as the positive control in this study. The limited survival of ceftazidime-treated mice was most likely due to the high *B. pseudomallei* challenge doses, which were approximately three times greater than targeted. This effect had been previously observed in our lab in other studies where higher challenge doses also occurred (20, 21). The observation that there were late deaths and that all tissues were colonized by *B. pseudomallei* at study termination is normal for this infection model (19, 22, 23), reflecting the fact that *B. pseudomallei* quickly colonizes intracellularly in other tissues beyond the lung, including the spleen and the liver, which are difficult to treat.

Encouragingly, the lack of deaths in the three LPC-233 treatment groups at dosing levels of 30 mg/kg q12h or higher until 15 days following the last dose indicates that LPC-233 had efficacy against acute *B. pseudomallei* infections represented by this model. Additionally, after the initial loss of body weight due to *B. pseudomallei* exposure, the mouse body weight recovered sooner in the LPC-233 treatment groups at 30 mg/kg q12h or higher compared to the ceftazidime group at 150 mg/kg q6h. Taken together, these results indicate that the LPC-233 treatment was more effective than ceftazidime under the same bacterial load and conditions, although we could not rule out the possibility that this difference reflected the less than optimal dosing regimen of ceftazidime due to its rapid turnover *in vivo*.

Even though our studies demonstrated superior effectiveness of LPC-233 over the frontline therapeutic ceftazidime in the intensive phase of the treatment of *B. pseudomallei* infection, the variation observed in bacteremia at the end of the 29-day observation period is a reflection of individual animals experiencing the end stage of disease, as bacteremia is usually only observed shortly before death. Hence, the treatment regimen of LPC-233 needs to be further optimized to overcome *B. pseudomallei* chronic infections.

LPC-233 demonstrated lung penetration with a prolonged time above the MIC in the infected PK study, but the pharmacodynamics driver for efficacy has yet to be defined. Therefore, it is possible that a single higher dose, multiple doses per day (e.g., from q12h to q6h), a longer dose duration (e.g., extending treatment from 14 days to 28–35 days), or some combination thereof could lead to better outcomes defined as enhanced survival after treatment cessation or complete clearance of infection.

Even though dose-limiting cardiovascular adverse effects have halted the development of advanced LpxC inhibitors, such as ACHN-975 (Achaogen) and RC-01 (Recida Therapeutics), LPC-233 does not appear to have these same liabilities (13). As LPC-233 is more potent than previous LpxC inhibitors (the MIC_90_ values for biothreat pathogens are 4–8 folds lower than any of the previous compounds, such as ACHN-975 (24)), has a lower clearance, and is orally available with a demonstrated efficacy, it is very likely that further optimization of the LPC-233 treatment regimen can lead to complete clearance of *B. pseudomallei* from infected mice.

## MATERIALS AND METHODS

### Bacterial strains and growth conditions

All *B. pseudomallei* strains evaluated in the susceptibility study are listed in Fig. S1 with the MIC data. The *B. pseudomallei* strain K96243 was obtained from BEI Resources (NR-4073). K96243 was initially isolated by Dr. Sirirurg Songsivilai in 1996 from a diabetic patient with fatal septicemic melioidosis in Thailand. The organism was obtained from BEI Resources and maintained as frozen stocks at the test facility (UFL-ITI, Orlando, FL). Antibiotic susceptibility determinations from stock strains yielded ceftazidime MIC of 2 µg/mL and an LPC-233 MIC_90_ of 0.03 µg/mL, as determined by the standard Clinical and Laboratory Standards Institute (CLSI) broth microdilution assay (14).

### MIC determination

The MIC testing for *B. pseudomallei* was conducted according to the CLSI method (25, 26). These were done in the cation-adjusted Mueller Hinton II broth (BBL, Becton Dickinson, Franklin Lakes, NJ), with incubation occurring at 35°C. The *Escherichia coli* strain ATCC 25922, *Pseudomonas aeruginosa* ATCC 27853, and *Staphylococcus aureus* strain ATCC 29213 were used as the antibiotic quality control strains according to the CLSI method. The results from these strains were read after approximately 18 h of incubation. All determinations were conducted in duplicate.

### Mice

Female BALB/c mice, which were 6 to 8 weeks old (average weight of ~20 g), were obtained from Charles River Laboratories, Kingston, NY. The animals were acclimated for 1 week prior to the aerosol challenge and had free access to food (Tekland LM-485 mouse/rat chow 7912 Harland) and water throughout the course of the study.

All experimental procedures described here were performed in accordance with a UF IACUC/ACURO approved protocol. We adhered to the guidelines promulgated in the *Guide for the Care and Use of Laboratory Animals* (27). The studies were conducted in the BSL-3 laboratories of the University of Florida, Orlando and complied with the Animal Welfare Act and other federal statutes and regulations relating to animals and experiments involving animals. The facility is fully accredited by the American Association for the Accreditation of Laboratory Animal Care.

### Weights

Mice were weighed by cage group immediately prior to the aerosol challenge, and then every two days throughout the study.

### Preparation of the *B. pseudomallei* challenge strain for aerosolization

*B. pseudomallei* K96243 (BEI resources NR-4073) was obtained through the National Institutes of Health (NIH) Biodefense and Emerging Infections Research Resources Repository, NIAID, NIH. The initial culture used for the animal challenges was started from a single-use frozen stock by plating on tryptic soy agar (TSA). Colonies from the stock plate were transferred to 10 mL of brain–heart infusion broth in a 125 mL flask and grown overnight at 35°C with shaking at 200 rpm. For the aerosol challenge, the overnight culture was adjusted to approximately 8 × 10^8^ CFU/mL based on the OD_600_ to deliver a target-challenge aerosol dose of approximately 25 × LD_50_ (LD_50_ = 76 CFU/mouse). To verify the final starting bacterial concentrations, the adjusted bacterial cultures were serially diluted and plated on TSA. The colonies were enumerated following overnight incubation of the plates at 35°C.

### Aerosol infection

A targeted inhaled dose of approximately 25 × LD_50_ of *B. pseudomallei* K96243 was administered to female BALB/c mice by whole-body aerosol exposure. Aerosols were generated using a three-jet Collison nebulizer (May 1973). All aerosol procedures were controlled and monitored using the automated bioaerosol exposure system (28) operating with a whole-body rodent exposure chamber. Integrated air samples were obtained from the chamber during each exposure using an all-glass impinger (AGI). The AGI collections were serially diluted and plated on TSA, incubated, and counted as described for the challenge strain enumeration. The *B. pseudomallei* inhaled dose (CFU/mouse) was estimated using mouse respiratory rates per Guyton’s formula (29) and calculated according to Roy (30).

### Infected PK

Single doses of LPC-233 were administered orally or IP, as indicated in Table 1 (0.1 mL/mouse). Doses were administered 48 h (treatment, not post-exposure prophylaxis) after the *B. pseudomallei* aerosol challenge. Four additional animals were challenged to euthanize at the time of antibiotic administration for the verification and quantification of infection by culture of the blood, lungs, and spleens.

Blood and ELF were collected from treated animals after CO_2_ asphyxiation at 0.25, 0.5, 1, 2, 4, and 6 h post-dosing. Blood samples were collected in K_2_-EDTA tubes. Plasma was collected by immediate centrifugation (3000×*g* for 5 min). Lung ELF was obtained by broncho-alveolar lavage (BAL) with physiological saline. To conduct the BAL, a small hole was cut in the trachea, distal to the lungs, then a blunt needle was inserted into the trachea and secured. Next, 0.5 mL was slowly injected and carefully withdrawn. Washing was repeated with a second 0.5 mL volume, and the samples were pooled. The combined volume yield was between 0.7 and0.9 mL.

The plasma and ELF samples were mixed with an equal volume of 100% methanol to sterilize any residual bacteria. A 10% vol of each treated sample was transferred to a methanol neutralizing media and incubated for 48 h to determine sterility. The remaining sample volumes were stored frozen at −80°C until sterilization was verified for removal from the BSL3 laboratory.

For each deactivated plasma sample, 20 µL was mixed with 10 µL of internal standard (IS) (LPC-087 5.00 µg/mL in acetonitrile:methanol, ACN:MeOH (1:1)) and 100 µL of ACN:MeOH (1:1), then vortexed well prior to centrifugation at 13.2 k rpm for 10 min. Approximately 100 µL of the supernatant was transferred to a microtiter plate and diluted with 100 µL of water prior to vortexing the plate. Subsequently, 1 µL of each sample was injected into the LC instrument for analysis. Standards, QC, and plasma sample concentrations represent a 2× dilution due to the dilution with the methanol (1:1, v:v). Sample concentrations were calculated with a 2× correction factor.

For each deactivated ELF sample, 100 µL was mixed with 10 µL of IS (LPC-087 5.00 µg/mL in ACN:MeOH (1:1)) and 100 µL of ACN:MeOH (1:1), then vortexed well prior to centrifugation at 21 k RCF for 10 min. Approximately 100 µL of the supernatants was transferred to a microtiter plate and diluted with 100 µL of water prior to vortexing the plate. Subsequently, 5 µL of each sample was injected for analysis. Standards, QC, and ELF sample concentrations represent a 2× dilution due to dilution with methanol (1:1, v:v). Sample concentrations were calculated with a 2× correction factor.

Urea concentrations in the plasma and ELF samples were measured and used to normalize the drug concentrations determined in the lavage solutions to account for the dilution factor in the ELF collection. Urea levels were determined by adding 20 µL of plasma or 100 µL of ELF samples (treated 1:1 with MeOH) to 10 µL of IS (Urea 15N2 100 µg/ml in water) and 100 µL of acetonitrile. Samples were vortexed then centrifuged at 32 k × RCF For plasma. Next, 180 µL of each supernatant was transferred into a well of a 96-well plate, and 10 µL was injected into the LC instrument for analysis. For ELF samples, 150 µL of the sample was transferred into a well of a 96-well plate, and 20 µL was injected into the LC instrument for analysis.

The LC–MS/MS instrument used for analysis was ABSciex API5000 operated in the negative ion mode using Waters Acquity BEH C18 2.1 × 50 mm, 1.7 µm at 40°C column. Injection volumes were as stated above with a flow rate of 0.4 mL/min 10% Mobile B, 90% Mobile A. Mobile phase A was 0.1% formic acid (FA) in water, while mobile phase B was 0.1% FA in ACN.

For urea quantification, the instrument used was API5000 with a Luna NH2 150 × 4.6, 5 µm column. The flow rate was 0.75 mL/min with isocratic 20% Mobile A, 80% Mobile B. Mobile phase A was 0.1% FA in water, while mobile phase B was 0.1% FA in ACN.

### Assessment of efficacy

The cohort size for statistical evaluation was 10 mice. Mortality was assessed and recorded every 6 h during the antibiotic administration phase of the study (14 days starting from 24 h post-challenge) and at least twice daily thereafter. The experiment was terminated at Day 43.

All surviving animals were euthanized. Blood was collected immediately by cardiac bleeds, and 100 µL was plated on TSA. Necropsy was performed to collect the lungs, spleens, and livers. Tissues were weighed and homogenized in 1 mL sterile saline, then serially diluted for spread plate enumeration (performed in duplicate on TSA). Cultures were incubated at 35°C to determine the bacterial load. For the lung and liver samples, the limit of detection was 50 CFU/total organ. The limits of detection for the spleen and blood were 5 × 10^3^ CFU/organ and 10 CFU/mL, respectively.

### Drugs and materials

LPC-233, Lot# 10757–005-002 (retest/expiration date: Nov. 2021) from Valanbio Therapeutics, Raleigh, North Carolina was shipped to University of Florida as a 30 mg/mL LPC-233 in 20% Captisol (Oral and Parenteral Solution) and stored at 4°C. For the desired dose concentrations of LPC-233, dilutions were made with the 20% Captisol vehicle. The prepared dosing formulations were stored protected from light at 4°C.

Ceftazidime Lot 110030c (expiration date: 2/2024) was procured from Sagent Pharmaceuticals, Schaumburg, Illinois. Accordingly, 5.8 mL of saline for injection (SI) was added to a 1 g vial (final volume: 6.6 mL), and the stock solution was diluted to a final concentration of 30 mg/mL with SI to create a dosing solution. Dosing solutions and syringes were prepared every 4 days and stored at 4°C protected from light.

Captisol (vehicle), ceftazidime, or LPC-233 doses were administered (Table 1) as a single 0.1 mL/mouse dose by oral gavage (PO) or intraperitoneally (IP) 24 h after the *B. pseudomallei* aerosol challenge.

### Data analysis

The area under the curve (log-trapezoid rule) 0–6 h, C_max_, and time above MIC were determined with Prism Version 6.05 (GraphPad Software). A big non-parametric adaptive grid (Big NPAG) was used for the population PK analysis. A set of four inhomogeneous differential equations was employed. the estimates of total drug in plasma AUC (AUC_PL_; 0–48 h) after the dose, AUC in ELF (AUC_ELF_; 0–48 h), penetration (AUC ratio: AUC_ELF_/AUC_PL_), free drug time > MIC in plasma (free fraction = 0.04, MIC_90_ = 0.03 mg/L), and time > MIC in ELF were calculated below using ADAPT 5 Package of Programs (31). This was performed as a single oral dose of 100 mg/kg at time zero. Multiple models were examined. A four-compartment model (absorption, central, peripheral, and ELF compartments) was chosen through both the likelihood ratio test and the Akaike information criterion. The ELF penetration was calculated as the ratio of AUC_48_ in ELF to AUC_48_ in plasma. In this instance, the calculations were performed with the median parameter vector, as this provided a fit of the model to the data that best reflected time > MIC (32). Since there were no measurements of protein binding in ELF, the values shown were total drug measurements.

### Analysis of LpxC and FabZ in *B. pseudomallei* strains

Translated sequences of LpxC and FabZ enzymes from the genomic DNA of *B. pseudomallei* strains H4609e, K96243, 406e, and H942dii were aligned and analyzed using Clustal Omega (33).

## SUPPLEMENTAL MATERIAL

Supplemental material aac.01295-24-s0001.docx Fig. S1 and S2; Table S1.

ASM does not own the copyrights to Supplemental Material that may be linked to, or accessed through, an article. The authors have granted ASM a non-exclusive, world-wide license to publish the Supplemental Material files. Please contact the corresponding author directly for reuse.
