# Supplementary material for: Evaluation of a potent LpxC inhibitor for post-exposure prophylaxis treatment of antibiotic-resistant Burkholderia pseudomallei in a murine infection model
Source: Antimicrob Agents Chemother. 2024 Dec 13;69(1):e01295-24. doi: 10.1128/aac.01295-24 (PMC11784359; doi:10.1128/aac.01295-24)
Supplement: Supplemental material — Fig. S1 and S2; Table S1. [file aac.01295-24-s0001.docx]

**Supplemental Material**

**Evaluation of a Potent LpxC Inhibitor for Post Exposure Prophylaxis Treatment of Antibiotic Resistant *Burkholderia pseudomallei* in a Murine Infection Model**

Henry S. Heine^1†^, Bret K. Purcell^1^, Clayton Duncan^2^, Lynda Miller^1^, John E. Craig^1^, Amanda Chase^1^, Lynne Honour^1^, Michael Vicchiarelli^1^, George L. Drusano^1^, and Pei Zhou^3†^

^1^Institute for Therapeutic Innovation, University of Florida, Orlando, Florida, USA

^2^Valanbio Therapeutics Inc., Raleigh, North Carolina, USA

^3^Department of Biochemistry, Duke University School of Medicine, Durham, North Carolina, USA

Running Title: LPC-233 PEP Treatment of *Burkholderia pseudomallei*

^†^Corresponding Authors.

Henry S. Heine, Mailing address: Institute for Therapeutic Innovation, University of Florida, 6550 Sanger Road, Orlando, Florida, 32827

Email: henry.heine@medicine.ufl.edu

Pei Zhou, Mailing address: Department of Biochemistry, Duke University School of Medicine, 303 Research Drive, Durham, North Caroline 27710

Email: peizhou@biochem.duke.edu

Keywords: melioidosis, *B. pseudomallei*, pneumonia, LpxC, LPC-233

**
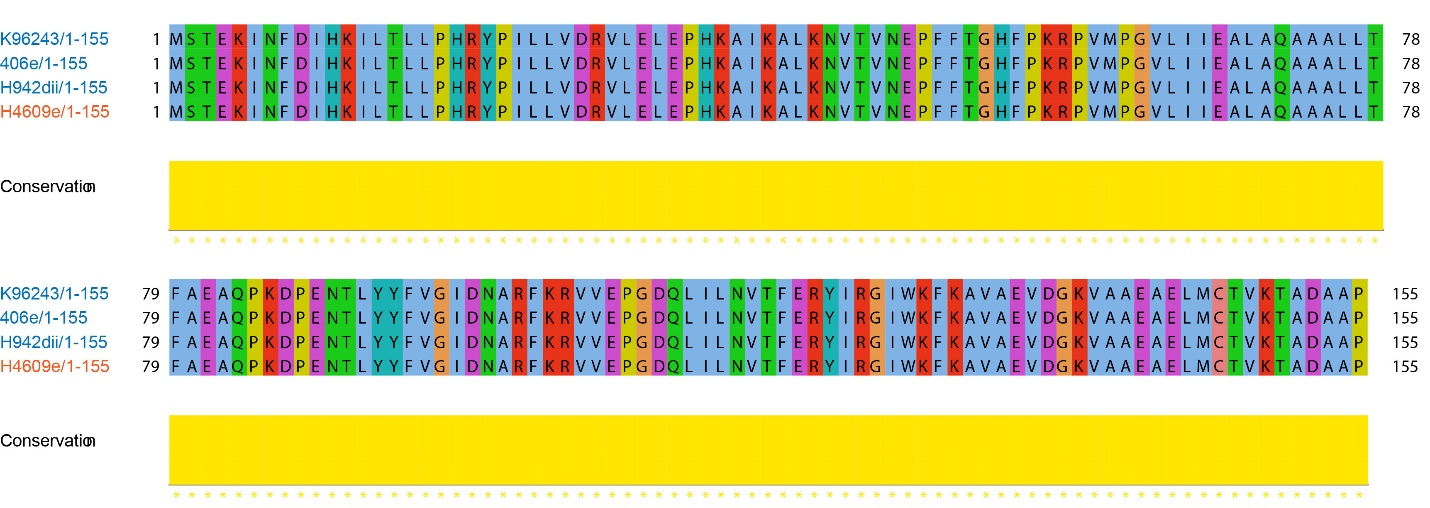
**

**Figure S1. Sequence comparison of the FabZ enzymes in *B. pseudomallei* strains.** Strains K96243, 406e, and H294dii (blue) are highly susceptible to LPC-233 with MIC values ≤ 0.008 μg/mL, whereas strain H4609e (orange) shows an elevated MIC of 0.12 μg/mL. Sequence alignment was conducted using Clustal Omega (33).

**
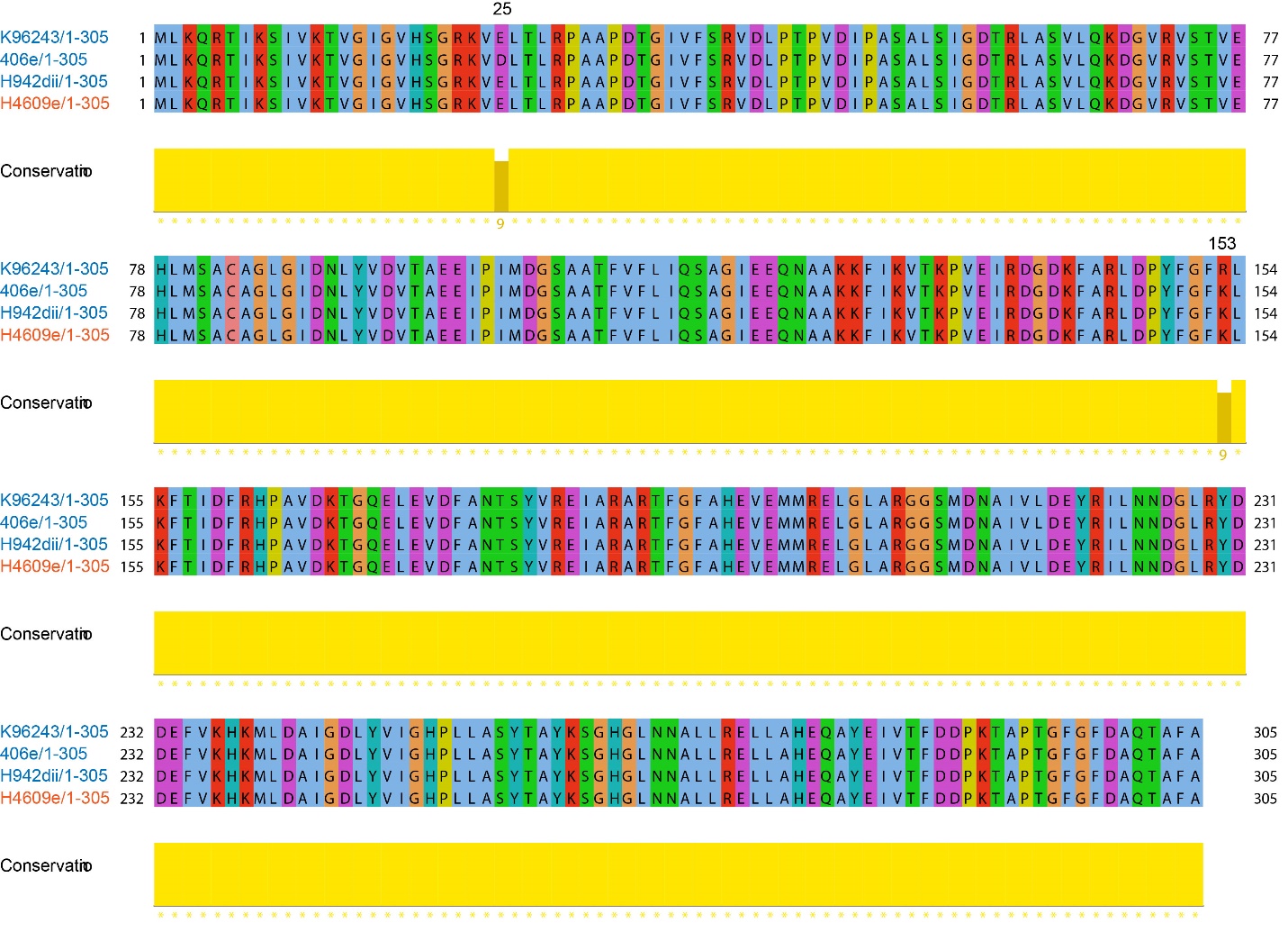
**

**Figure S2. Sequence comparison of the LpxC enzymes in *B. pseudomallei* strains.** LpxC enzymes show naturally occurring polymorphism at position 25 (E/D) and 153 (K/R). Strains K96243, 406e, and H294dii (blue) are highly susceptible to LPC-233 with MIC values ≤ 0.008 μg/mL, whereas strain H4609e (orange) shows an elevated MIC of 0.12 μg/mL. Sequence alignment was conducted using Clustal Omega (33).

**Table S1: MICs for 30 strains of *Burkholderia pseudomallei* (concentration: µg/mL)**

| **Strain** | **LPC-233** | **LPC-233** | **Ceftazidime** |
| --- | --- | --- | --- |
| H5598c | ≤0.008 | ≤0.008 | 2 |
| H316c | 0.03 | 0.03 | 4 |
| H942dii | ≤0.008 | ≤0.008 | >16 |
| H979bii | ≤0.008 | ≤0.008 | 0.5 |
| H4609e | 0.12 | 0.12 | >16 |
| Bp 0537 RF43-Bp22 | ≤0.008 | ≤0.008 | 8 |
| Bp 3994 MSHR730 | ≤0.008 | ≤0.008 | 2 |
| Bp 4002 MSHR296 | ≤0.008 | ≤0.008 | 2 |
| Bp 4003 MSHR840 | ≤0.008 | ≤0.008 | 0.5 |
| Bp 4075 NAU21B9 | ≤0.008 | ≤0.008 | 2 |
| Bp 4160 MSHR1043 | ≤0.008 | ≤0.008 | 2 |
| Bp 1829 INT2-Bp92 | ≤0.008 | ≤0.008 | 2 |
| W28405-3/Bp1651 | ≤0.008 | ≤0.008 | 4 |
| 1710a | 0.03 | 0.03 | >16 |
| 1106a | ≤0.008 | ≤0.008 | 2 |
| 406e | ≤0.008 | ≤0.008 | 2 |
| 576a | ≤0.008 | ≤0.008 | 2 |
| MSHR491 | ≤0.008 | ≤0.008 | 2 |
| MSHR465a | ≤0.008 | ≤0.008 | 2 |
| 2661a | 0.03 | 0.03 | 2 |
| E0024 | ≤0.008 | ≤0.008 | 2 |
| E0181 | ≤0.008 | ≤0.008 | 2 |
| E0241 | ≤0.008 | ≤0.008 | 2 |
| E0371 | ≤0.008 | ≤0.008 | 2 |
| PHLSSS83 | ≤0.008 | ≤0.008 | 1 |
| NCTC4845 | ≤0.008 | ≤0.008 | 2 |
| K96243 | ≤0.008 | ≤0.008 | 2 |
| Pasteur 6068 | ≤0.008 | ≤0.008 | 2 |
| S13 | ≤0.008 | ≤0.008 | 2 |
| 1026b | 0.03 | 0.03 | 2 |
| **Range** | ≤0.008 - 0.12 | | 0.5 - >16 |
| **MIC_50_** | ≤0.008 | | 2 |
| **MIC_90_** | 0.03 | | 8 |
| **Quality Control Strains** | | | |
| *Escherichia coli* ATCC 25922 | ≤0.008 | ≤0.008 | 0.12 |
| *Pseudomonas aeruginosa* ATCC 27853 | 0.25 | 0.25 | 1 |
| *Staphylococcus aureus* ATCC 29213 | >16 | >16 | 8 |
